# Molecular mimicry of NMDA receptors may contribute to neuropsychiatric symptoms in severe COVID-19 cases

**DOI:** 10.1186/s12974-021-02293-x

**Published:** 2021-10-28

**Authors:** Veronika Vasilevska, Paul C. Guest, Hans-Gert Bernstein, Matthias L. Schroeter, Christian Geis, Johann Steiner

**Affiliations:** 1 Laboratory of Translational Psychiatry, Department of Psychiatry and Psychotherapy, Otto-Von-Guericke-University Magdeburg, Leipziger Str. 44, 39120 Magdeburg, Germany; 2 Laboratory of Neuroproteomics, Department of Biochemistry and Tissue Biology, University of Campinas (UNICAMP), Campinas, Brazil; 3 Department of Neurology, Max Planck Institute for Human Cognitive and Brain Sciences, Clinic for Cognitive Neurology, University Hospital Leipzig, Leipzig, Germany; 4 Section Translational Neuroimmunology, Department of Neurology, Jena University Hospital, Jena, Germany; 5 German Center for Mental Health (DZP), Center for Intervention and Research On Adaptive and Maladaptive Brain Circuits Underlying, Mental Health (C-I-R-C), Jena-Magdeburg-Halle, Germany; 6 Center for Behavioral Brain Sciences, Magdeburg, Germany

**Keywords:** SARS-CoV-2, COVID-19, Inflammation, Autoimmune encephalitis, NMDA receptor, Corticosteroid, Immunomodulatory agent

## Abstract

Approximately 30% of individuals with severe SARS-CoV-2 infections also develop neurological and psychiatric complaints. In rare cases, the occurrence of autoimmune encephalitis has been reported after SARS-CoV-2 infection. In this systematic review, we have identified eight SARS-CoV-2-associated cases of anti-NMDA receptor encephalitis. All had cerebrospinal fluid antibodies against the NMDA receptor and a recent onset of working memory deficits, altered mental status, or psychiatric symptoms, such as confusion, agitation, auditory hallucination, catatonia and speech dysfunction. All patients received high-dose steroid and immunoglobulin therapeutics and conditions improved in each case. These findings suggest that clinical attention should be paid to warning signs of autoimmune encephalitis in severe COVID-19 cases. If characteristic features of autoimmune encephalitis are present, autoantibody diagnostics should be performed and confirmed cases should be treated with immunotherapy to minimize neurological impairments.

**Supplementary Information:**

The online version contains supplementary material available at 10.1186/s12974-021-02293-x.

## Introduction

The COVID-19 pandemic caused by the SARS-CoV-2 virus has now affected more than 2% of the world population with over 185 million cases and 4 million deaths [1]. In addition to effects on health and mortality, the spread of the virus across the globe as well as governmental responses to the pandemic have had dire effects on human contacts and global economies and cases of anxiety and depression have increased in parallel, even in the non-infected population [2]. More directly, one study showed that approximately 30% of those infected by the virus and who experienced a severe course of the disease also developed psychological complaints, such as post-traumatic stress disorder (PTSD) [3]. Another study showed that of 125 severe cases registered as part of the CoroNerve study with neurological and psychiatric presentations of COVID-19 infection, 39 (31%) presented with altered mental status and 23 (18%) of these fulfilled the clinical case definitions for psychiatric disorders including new-onset psychosis, neurocognitive syndrome and affective disorder [4].

Yapici-Eser and colleagues recently described a potential pathomechanism based on molecular mimicry that may contribute to development of COVID-19-associated neuropsychiatric symptoms [5]. Structural similarities between the *N*-methyl-d-aspartic acid receptor (NMDAR) GluN1 (synonym NR1) and GluN2a (synonym NR2a) subunits with the SARS-CoV-2 nonstructural protein 8 (NSP8) and 9 (NSP9), respectively, may induce immune-mediated cross-reactivity to the NMDAR. These proteins are essential for replication of the virus and can interact directly with glutamate receptors of the NMDA and metabotropic families, leading to changes in membrane resting-state and action potentials [5]. Molecular mimicry may lead to generation of immunoglobulin G (IgG) antibodies against the NMDAR after SARS-CoV-2 infections. Anti-NMDAR encephalitis, mediated by IgG antibodies to the GluN1 subunit, is a common form of autoimmune encephalitis characterized by presentation of neurological and psychosis-like symptoms [6]. In this disease, antibodies bind to the NMDAR, induce crosslinking and receptors are subsequently internalized and thus are no longer available for excitatory glutamatergic transmission [6]. Viral diseases have been identified as potential triggers. For instance, anti-NMDAR-encephalitides can occur as a secondary disease after infection with viruses such as herpes simplex 1 or varicella zoster [7]. Moreover, past influenza A or B infections were identified as predisposing factors for NMDAR autoantibody seropositivity [8]. Accordingly, we hypothesized that SARS-CoV-2 might similarly induce anti-NMDAR encephalitis as a direct consequence of infection or secondarily through subsequent activation of autoimmune processes. Viruses such as SARS-CoV-2 hijack the cellular machinery of the host cell in order to reproduce themselves. In the process, their mimicry of key motifs of host proteins can lead to disruption of vital cellular functions, activate inflammation pathways and alter the immune response [9].

Here, we aimed to identify published cases of anti-NMDAR encephalitis with concurrent neuropsychiatric symptoms temporally associated with SARS-CoV-2 infections.

## Methods

**Fig. 1:**
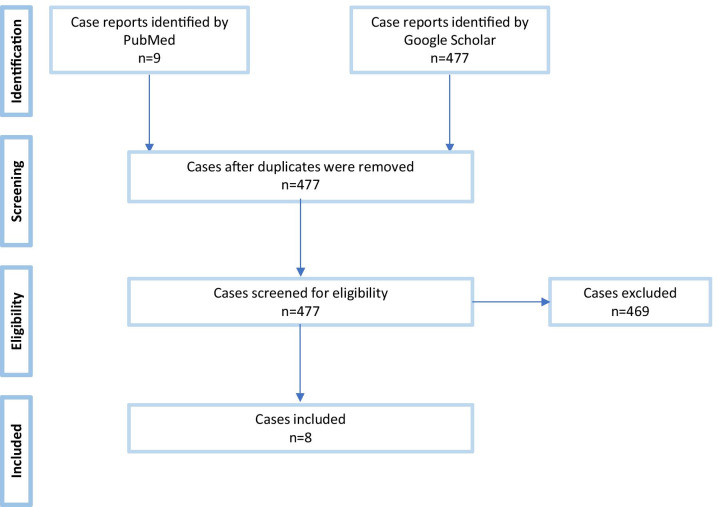
Selection procedure of the case reports. After reading of titles and abstracts, 469 publications could be excluded out of 477 potentially relevant articles, resulting in eight relevant cases finally. One unpublished congress poster matching search criteria was excluded due to the impossibility to perform a qualitative check (https://www.charcot-ms.org/files/Annual-Meetings/28/Abstracts-2020/42_ECF2020_Abstract_CL_Ramos_E.pdf)

We searched the PubMed and Google Scholar databases using the search terms “NMDA encephalitis” or “NMDAR encephalitis” or “NMDA receptor encephalitis” and “SARS-CoV-2” or “COVID-19” to identify relevant cases. The last search was performed on September 20th 2021. Preferred Reporting Items for Systematic Reviews and Meta-Analyses (PRISMA, http://prisma-statement.org) were applied. The process for selecting studies (identification, screening, eligibility and inclusion in the systematic review) is reported in a flow diagram in Fig. 1. Studies were checked for eligibility and selected by two persons (VV and PCG). Initially, many more papers were identified in Google Scholar than in PubMed. However, most of these articles did not meet the search criteria as, on close inspection, they were comments on already published articles, meta-analyses, etc. For the final evaluation, only case reports or case series in original reports published in peer-reviewed international journals were considered. Articles in languages other than English or German were translated with the help of DeepL Translator (https://www.deepl.com). Finally, only 7 English [10–16] and one Spanish article [11] met the search criteria. The quality of the identified case reports was checked according to the CARE Case Report Guidelines (2013 CARE Checklist: www.care-statement.org) as summarized in Additional file 1: Table S1. Cases were included if they were positive for SARS-CoV-2 in nasopharyngeal swab or blood or cerebrospinal fluid (CSF) tests and suffered from anti-NMDAR encephalitis as defined by Graus et al. [17]. We extracted information regarding patient age and medical history, reason for hospitalization, respiratory symptoms, neuropsychiatric symptoms, blood-based biomarkers, magnetic resonance imaging (MRI) of the brain, CSF biomarkers and electroencephalography (EEG) readings, as well as therapies attempted and clinical outcomes, as available.

## Results

We identified eight case reports in which anti-NMDAR encephalitis (characteristic disease symptoms and detection of NMDAR antibodies in CSF) occurred with a time delay of 3 days to 3 weeks after the manifestation of COVID-19 disease (Table 1). Four of the identified 8 patients developed symptoms with a time delay of less than one week. The age of the patients ranged from 23 months to 53 years and consisted of four males and four females. All patients had positive GluN1 antibodies in the CSF. Only patients 1–4 showed pronounced respiratory symptoms including low O_2_ saturation, bilateral milk glass opacities on chest x-ray and patchy bibasilar consolidation on chest computed tomography (CT) imaging [10–12], and severe hypoxemia with bilateral infiltrations [13]. Patients 2, 3 and 5–8 developed epileptic seizures during the course [11, 12, 14–16, 18]. All patients had psychiatric or neurological symptoms, manifested as a disturbance of consciousness, delirium, psychosis or catatonia. Patients 1 and 5–8 exhibited motor failures or dysarthria [10, 14–16, 18]. Cases 4 and 8 had increased levels of C-reactive protein (CRP) and patients 3, 4 and 8 had decreased lymphocytes [12, 13, 16]. Increased interleukin-6 (IL-6) levels were found in blood and CSF in one subject (patient 5) [14] and only in CSF in another (patient 1) [10]. Patients 1–6 had elevated lymphocytes in CSF [10–15]**.** Cases 3 and 8 were SARS-CoV-2 positive in the CSF [12, 16]. Patient 2 had ovarian teratoma as an associated malignancy and potential alternative trigger of disease [11]. EEG recordings from patients 1, 4, 5 and 8 showed a slowdown, and one of these (patient 5) showed an extreme delta brush-like pattern, typical for the diagnosis [10, 13, 14, 16]. In five patients, imaging examinations of the brain appeared normal but patient 4 showed hyperintensities on fluid-attenuated inversion recovery (FLAIR) images in left amygdala, left anterior putamen with negligible effects in the right amygdala [13]. It should be noted that in this latter case, an initial SARS-CoV-2 swab PCR test was negative, raising the possibility that the infection may have been hospital-acquired. Each patient received guideline-compliant steroid therapy with intravenous immunoglobulins, patients 5 and 8 underwent plasmapheresis [14, 16], and conditions improved in all cases.

**Table 1:** Summary of COVID-19 cases identified involving anti-NMDAR encephalitis with neuropsychiatric symptoms

Patient 1	Panariello et al., 2020 [10]	Male (23 years-old). History drug abuse
	Reason for hospitalization	Psychomotor agitation, anxiety, formal thought disorder, persecutory delusions and auditory hallucinations and global insomnia. SARS-CoV-2 positive nasopharyngeal swab RT-PCR testing
	Respiratory (other) symptoms	Fever, drop in O_2_ saturation, chest X-ray: bilateral milk glass opacities, chest CT: patchy bibasilar consolidation
	Neuropsychiatric symptoms	Confusion, disorganization of speech, thought/behaviour, auditory hallucinations and insomnia. Week 2: mutistic/non-responsive. Week-3: dysphagia, dyskinesia, autonomic instability, fluctuations in body temperature, blood pressure, pulse and respiratory rate
	Blood test	IL-6 not mentioned, no lymphopenia at anti-NMDAR encephalitis diagnosis, hyponatremia
	CSF examination	SARS-CoV-2 negative. IL-6 elevated, NMDAR-antibodies positive. Virological and microbiological diagnostics negative. Elevated red and white cells
	EEG	Theta activity, unstable, non-reactive to visual stimuli
	Therapy	Seizure prophylaxis. No symptom improvement with antipsychotics. COVID-19 therapy with hydroxychloroquine and darunavir/cobicistat. Antibiotic prophylactic therapy. After anti-NMDAR encephalitis diagnosis, dexamethasone and intravenous immunoglobulin
	Course	Clinical symptoms improved
Patient 2	Alvarez Bravo and Ramio, 2020 [11]	Female (30 years-old). No previous medical history. SARS-CoV-2 positive nasopharyngeal swab RT-PCR testing
	Reason for hospitalization	Behavioral changes
	Respiratory (other) symptoms	Fever, pneumonia, thrombosis of the left iliac vein, and bilateral pulmonary embolism attributed to SARS-CoV-2 infection (Ovarian teratoma)
	Neuropsychiatric symptoms	Psychomotor agitation, paranoid ideation, dysarthria with dysprosody, and visual hallucinations, focal and generalised seizures
	Blood test	SARS-CoV-2 positive
	CSF examination	Cells count and protein elevated. SARS-CoV-2 negative, NMDAR antibodies positive. Virological and microbiological diagnostics negative
	EEG	Epileptic discharges in the left frontotemporal region
	Therapy	After anti-NMDAR encephalitis diagnosis, 5 days of methylprednisolone and immunoglobulins administered
	Course	Hypoprosexia, emotional lability and memory disorder, Stabilised systemic and respiratory symptoms
Patient 3	Allahyari et al., 2021 [12]	Female (18 years-old). No previous medical history. SARS-CoV-2 positive nasopharyngeal swab RT-PCR testing
	Reason for hospitalization	Generalized tonic–clonic seizures
	Respiratory (other) symptoms	Fever, pneumonia, hypotonia, tachycardia, tachypnea, oxygen saturation of 90%, bilateral pulmonary crackles in lower lung zones,
	Neuropsychiatric symptoms	3-week history of mood change as depression and anhedonia accompanied by lack of concentration, generalized tonic–clonic seizures
	Blood test	Neutrophilia, lymphopenia, CRP normal
	CSF examination	Cells count elevated. SARS-CoV-2 positive, NMDAR antibodies positive. Virological and microbiological diagnostics negative
	EEG	Epileptic discharges in the left frontotemporal region
	Therapy	Seizure prophylaxis. COVID-19 therapy with Remdesivir, Lopinavir/Ritonavir, and Interferon b1a (Resigen). Antibiotic prophylactic therapy. After anti-NMDAR encephalitis diagnosis, methylprednisolone and intravenous immunoglobulin
	Course	After 2 months of hospitalization discharged with full recovery
Patient 4	McHattie et al., 2021 [13]	Female (53 years-old). Ductal carcinoma of breast in remission. History of depression and psoriasis. Medications: sertraline, ciclosporin
	Reason for hospitalization	2-week confusion, fever and myalgias. SARS-CoV-2 negative on admission, positive on day-14 in nasopharyngeal swab RT-PCR testing
	Respiratory (other) symptoms	Severe hypoxemia with O2 dependency. Chest X-ray: bilateral infiltrations
	Neuropsychiatric symptoms	Day-5: catatonic symptoms of severe echolalia, palilalia, perseverations and echopraxia. Speech high-pitched and behavioural disinhibition. Left-side discrete hemiparesis, non-responsive to commands. Day-17: focal seizures, marked dysautonomia (increasingly hypotensive with bradycardia). Hyperkinetic movement disorder not present
	Blood test	CRP elevated with lymphopenia. NMDAR antibodies negative
	CSF examination	SARS-CoV-2 negative. Leukocytes high. Low glucose and high protein. Virological and microbiological diagnostics negative. NMDAR antibodies positive
	EEG	Slow activity on admission. No evidence of epileptiform discharges
	Therapy	Antiepileptic treatment. For suspected viral encephalitis, initial therapy with aciclovir and steroids. COVID-19 therapy with hydroxychloroquine, antibacterial and antifungal treatment. After anti-NMDAR encephalitis diagnosis, steroids, intravenous immunoglobulins and tocilizumab
	Course	Worsening symptoms with steroids. 1-month therapy: neuropsychiatric symptoms improved but persistence of left-side weakness. Cardiac MRI day 70: regression of signal changes. Brain MRI: atrophy of left amygdala and left hippocampus
Patient 5	Monti et al., 2020 [14]	Male (50 years-old). Moderate arterial hypertension
	Reason for hospitalization	Acute psychiatric symptoms. SARS-CoV-2 positive nasopharyngeal swab RT-PCR testing
	Respiratory (other) symptoms	None. No diarrhoea. Fever present
	Neuropsychiatric symptoms	Confabulations and delirium. Day-4: focal motor seizures with reduced consciousness, orofacial dyskinesia, automatisms. Sudden refractory status epilepticus
	Blood test	IL-6 elevated. No CRP elevation or leukocytosis
	CSF examination	SARS-CoV-2 not mentioned. Third lumbar puncture: NMDAR antibodies positive, cell count and IL-6 elevated. Oligoclonal bands positive. Virological and microbiological diagnostics negative
	EEG	Diffuse delta activity with extreme delta brush pattern.Anterior subcontinuous periodic theta activity
	Therapy	Antiepileptics and anaesthetics. COVID-19 therapy with hydroxychloroquine and lopinavir/ritonavir. After diagnosis of anti-NMDAR encephalitis: corticosteroids, immunoglobulins and plasmapheresis
	Course	4 months after symptom onset patient discharged in good condition with no neuropsychiatric symptoms
Patient 6	Burr et al., 2021 [15]	Female (23 months-old). Vaccinated. No previous diseases. Family history unremarkable
	Reason for hospitalization	Fever, psychomotor agitation, sleep disturbances, constipation, decreased oral intake. SARS-CoV-2 positive nasopharyngeal swab RT-PCR testing
	Respiratory (other) symptoms	None. Fever, dehydration present
	Neuropsychiatric symptoms	Agitation, poor sleep, mood swings, mutism, regular kicking/ flapping of extremities. Day-2: multiple epileptic seizures. Week 2: worsening encephalopathy with persistent hyperkinetic movements of extremities and head
	Blood test	CRP normal, NMDAR antibodies positive, IL-6 not mentioned
	CSF examination	SARS-CoV-2 negative. Mild elevation of leukocytes. Oligoclonal bands negative. Virological and microbiological diagnostics negative. NMDAR antibodies positive. IL-6 not mentioned
	EEG	Not mentioned
	Therapy	Antiepileptics. After anti-NMDAR encephalitis diagnosis, corticosteroid therapy for 5 days with no improvement, followed by intravenous immunoglobulin administration
	Course	Remission within one week after immunoglobulin therapy
Patient 7	Sanchez-Morales et al., 2021 [18]	Male (14 years-old). No previous medical history. SARS-CoV-2 positive nasopharyngeal swab RT-PCR testing
	Reason for hospitalization	Behavioral changes and neurological symptoms
	Respiratory (other) symptoms	None
	Neuropsychiatric symptoms	Altered behaviour and mental status, epileptic seizures, insomnia, orolingual dyskinesia
	Blood test	SARS-CoV-2 negative
	CSF examination	SARS-CoV-2 positive, NMDAR antibodies positive. Virological and microbiological diagnostics negative
	EEG	Not mentioned
	Therapy	After anti-NMDAR encephalitis diagnosis, methylprednisolone and immunoglobulins administered
	Course	Complete remission of neurological impairment. Control of epilepsy. Persistence of psychiatric symptoms
Patient 8	Sarigecili et al., 2021 [16]	Male (7 years-old). Vaccinated. No previous diseases. No abnormal family history
	Reason for hospitalization	Gait disorder. SARS-CoV-2 positive nasopharyngeal swab RT-PCR testing
	Respiratory (other) symptoms	None. No headache, fever, or cold symptoms. Day 8: tachycardia
	Neuropsychiatric symptoms	Ataxia and broad-based gait with poor muscle reflexes. Day-2: somnolence and epileptic seizures. Day 8: choreiform movements of extremities, tongue protrusion, bruxism, smacking, psychomotor agitation, catatonia, echolalia
	Blood test	CRP elevated, lymphopenia. IL-6 not mentioned
	CSF examination	No cells present. Oligoclonal bands negative. Virological and microbiological diagnostics negative. NMDAR antibodies positive. IL-6 not mentioned
	EEG	Encephalopathic pattern with disseminated delta waves
	Therapy	Antiepileptics after onset of seizures. Initial therapy with antibiotics/antivirals. After diagnosis of anti-NMDAR encephalitis: plasmapheresis three times, corticosteroid 7 days, immunoglobulins 5 days followed by corticosteroid again
	Course	Day 31: patient discharged walking but mildly ataxic with prednisolone and antiepileptic treatment. Possibility of repeat immunoglobulin administration

## Discussion

**Fig. 2:**
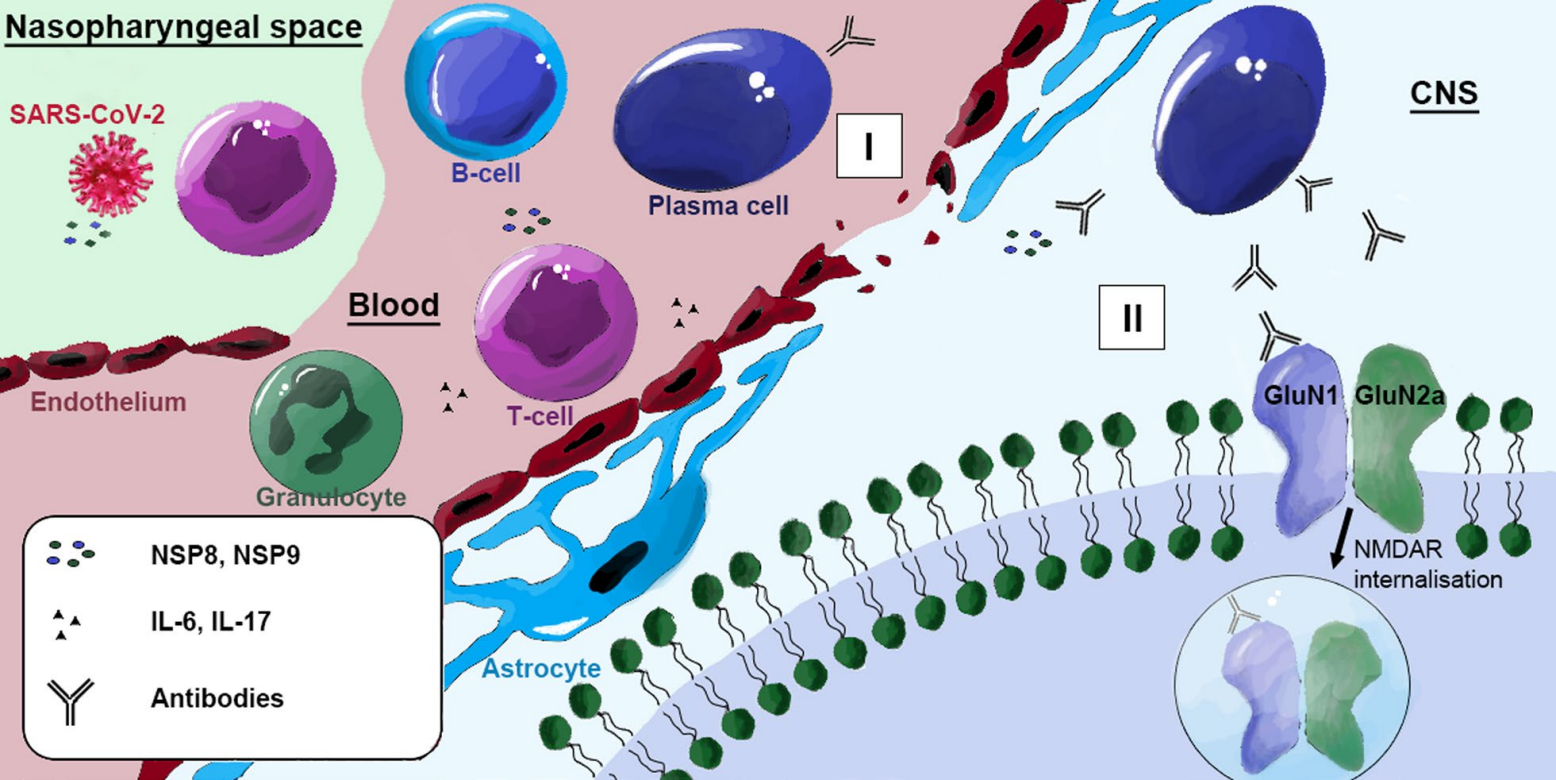
Possible pathophysiology of anti-NMDAR encephalitis induced by SARS-CoV-2. During acute COVID-19 infection, viral particles, including NSP8 and NSP9, are released. The released proteins are recognized by T cells, leading to activation of B cells, which become plasma cells and produce IgM and later IgG antibodies against NSP8 and NSP9. I SARS-CoV-2-associated endothelitis and IL-17 produced by activated T cells disrupt the blood–brain barrier, allowing NMDAR antibodies to enter the CNS. IL-6 alters glial activity and initiates neutrophil granulocyte migration leading to further blood-brain barrier destruction and inflammation. II Due to molecular mimicry, antibodies produced by plasma cells in the CNS can cross-react with the NMDAR subunits GluN1, leading to receptor internalization with subsequent degradation. Annotation: This schematic illustration does not correspond to the natural size proportions

This is the first systematic review to demonstrate a potential link between SARS-CoV-2 infection and secondary occurrence of anti-NMDAR encephalitis presenting with characteristic neuropsychiatric disorders. It is possible that mimicry of non-structural proteins of the SARS-CoV-2 virus with NMDA receptor subunit epitopes may have been the underlying cause of the autoimmune response against brain NMDA receptors (Fig. 2). However, further studies are required to pinpoint the precise mechanism of how such effects of the virus lead to central nervous system (CNS) autoimmunity.

IL-6 and Th17 cells appear to be involved in the pathophysiology of COVID-19-associated neuropsychiatric symptoms [19, 20] (Fig. 2). IL-6 is a key proinflammatory cytokine that can alter neuronal and glial activity and induce axonal degeneration [21, 22]. Moreover, IL-6 levels correlate with the severity of disease course in COVID-19 [23]. IL-6-dependent Th17 activation and differentiation appears to be essential for neutrophil granulocyte migration [23]. Consequently, the proinflammatory cytokine IL-17 produced by Th17 CD4 + T cells may disrupt the blood–brain barrier (BBB) and contribute to the strong association of Sars-Cov-2 with a variety of different secondary autoimmune diseases (e.g. systemic lupus erythematosus, Guillain-Barré syndrome, antiphospholipid syndrome, large vessel vasculitis and thrombosis, psoriasis, inflammatory arthritis, autoimmune thyroiditis, type-I-diabetes) and circulating autoantibodies (e.g., anti-nuclear antibodies/ANA, anti-cardiolipin/aCL antibodies, cytoplasmic and perinuclear anti-neutrophil cytoplasmic antibodies/cANCA and pANCA, antiprothrombin IgM) [24]. Accordingly, Sars-Cov-2 has been referred to as an "autoimmune virus" [24].

Alternatively, microvascular damage caused during the systemic inflammatory response to SARS-CoV-2 infection may promote development of encephalopathy in severe cases [21] (Fig. 2). Inflammatory mediators produced by the alveolar epithelium, macrophages, and leukocytes may contribute to endothelial inflammation, increased vascular permeability, edema, and increased turnover of coagulation factors. The increased vascular permeability can also cause disturbances of the microcirculation including BBB impairment [21]. This disruption of the BBB gives rise to another possible mechanism involving facilitated transfer of NMDAR-directed antibodies, into the CNS [25].

This study was limited by the small number of patients identified with SARS-CoV-2-related autoimmune anti-NMDAR encephalitis. In addition, an aetiological role of SARS-CoV-2 in the generation of anti-NMDAR encephalitis is not definitive. In one of the cases an initial SARS-CoV-2 swab was negative and the patient only tested positive after the onset of anti-NMDAR symptoms [13]. In another case, an ovarian teratoma was present which is a potential alternative trigger of anti-NMDAR encephalitis [11]. However, more carefully-characterised studies should become available as the pandemic progresses and eventually recedes. This is important as COVID-19 patients with severe infections might have a higher risk for developing neurological manifestations such as autoimmune encephalitis. This highlights the importance of appropriate screening of patients using serological and CSF assessment via cell-based-assays for antineuronal antibodies, as well as brain imaging and EEG recordings [26]. Potentially, one might also include biomarkers for glial or neuronal damage such as S100B, myelin basic protein, neurofilament light chain (NfL), or neuron-specific enolase to screen for brain alterations [27–29]. Furthermore, it is important to monitor patients for neurological warning signs of autoimmune encephalitis including seizures, personality and memory changes, psychotic symptoms, delusional thinking, headache, dizziness, catatonia and dyskinesias [17, 30, 31].

The eight cases of anti-NMDAR encephalitis detailed in this paper also calls attention to the increased risk associated with the sudden onset or intensification of neuropsychiatric symptoms. All of the autoimmune encephalitis patients described in this study improved following high-dose steroids and immunoglobulin therapy, thus highlighting the importance of early immunotherapy once the diagnosis of autoimmune encephalitis has been made.

## Supplementary Information

Below is the link to the electronic supplementary material.

**Additional file 1. Table S1.** The quality of the identified case reports has been checked according to the CARE Case Report Guidelines (www.care-statement.org). For patient 7, this check was not feasible, because this case came from a retrospective original report: Sanchez-Morales et al., 2021 [18].

## Data Availability

Not applicable.

## Abbreviations

aCL: Anti-cardiolipin antibody
ANA: Anti-nuclear antibody
BBB: Blood–brain barrier
cANCA: Cytoplasmic anti-neutrophil cytoplasmic antibody
CD4 + T cells: Cluster of differentiation 4 expressing lymphocytes matured in the thymus
CNS: Central nervous system
COVID-19: Coronavirus disease 2019
CRP: C-reactive protein
CSF: Cerebrospinal fluid
CT: Computed tomography
EEG: Electroencephalography
FLAIR: Fluid-attenuated inversion recovery
GluN1: *N*-Methyl-d-aspartic acid receptor subunit 1
GluN2a: *N*-Methyl-d-aspartic acid receptor subunit 2a
IgG: Immunoglobulin G
IgM: Immunoglobulin M
IL: Interleukin
MRI: Magnetic resonance imaging
NfL: Neurofilament light chain
NMDA: *N*-Methyl-d-aspartic acid
NMDAR: *N*-Methyl-d-aspartic acid receptor
NR1: *N*-Methyl-d-aspartic acid receptor subunit 1
NR2a: *N*-Methyl-d-aspartic acid receptor subunit 2a
NSP8: Nonstructural protein 8
NSP9: Nonstructural protein 9
O_2_: Oxygen
pANCA: Perinuclear anti-neutrophil cytoplasmic antibody
PTSD: Post-traumatic stress disorder
PubMed: Search engine accessing primarily the MEDLINE (Medical Literature Analysis and Retrieval System Online) database of references and abstracts on life sciences and biomedical topics, maintained by the United States National Library of Medicine (NLM) at the National Institutes of Health
SARS-CoV-2: Severe acute respiratory syndrome coronavirus type 2
Th17 cells: T helper cells producing interleukin 17
