# Supplementary material for: Molecular mimicry of NMDA receptors may contribute to neuropsychiatric symptoms in severe COVID-19 cases
Source: J Neuroinflammation. 2021 Oct 28;18:245. doi: 10.1186/s12974-021-02293-x (PMC8551937; doi:10.1186/s12974-021-02293-x)
Supplement: Supplementary file 1 — Additional file 1. Table S1. The quality of the identified case reports has been checked according to the CARE Case Report Guidelines (www.care-statement.org). For patient 7, this check was not feasible, because this case came from a retrospective original report: Sanchez-Morales et al., 2021 [18]. [file 12974_2021_2293_MOESM1_ESM.docx]

**Table S1**: The quality of the identified case reports has been checked according to the CARE Case Report Guidelines ([www.care-statement.org](http://www.care-statement.org)). For patient 7, this check was not feasible, because this case came from a retrospective original report: Sanchez-Morales et al., 2021 [18].

| **Item Nr.** | **Item Name** |  | **Patient 1**  Panariello et al., 2020 [10] | **Patient 2**  Alvarez Bravo, Ramio, 2020 [11] | **Patient 3**  Allahyari et al., 2021 [12] | **Patient 4**  McHattie et al., 2021 [13] | **Patient 5**  Monti et al., 2020 [14] | **Patient 6**  Burr et al., 2021 [15] | **Patient 8**  Sarigecili et al., 2021 [16] |
| --- | --- | --- | --- | --- | --- | --- | --- | --- | --- |
| 1 | Title | *The diagnosis or intervention of primary focus followed by the words “case report”.* | + | - | + | - | - | - | - |
| 2 | Key Words | *2 to 5 key words that identify diagnoses or interventions in this case report (including "case report").* | + | - | + | - | + | + | + |
| 3 | Abstract  (structured or unstructured) | *Introduction – What is unique about this case and what does it add to the scientific literature?*  *The patient’s main concerns and important clinical findings.*  *The primary diagnoses, interventions, and outcomes.*  *Conclusion – What are one or more “take-away” lessons from this case report?* | -  -  -  - | -  -  -  - | +  +  +/-  - | -  -  -  - | -  -  -  - | -  -  -  - | +  +  +  + |
| 4 | Introduction | *Briefly summarizes why this case is unique and may include medical literature references.* | + | + | - | + | + | + | + |
| 5 | Patient Information | *De-identified patient specific information.*  *Primary concerns and symptoms of the patient.*  *Medical, family, and psychosocial history including relevant genetic information.*  *Relevant past interventions and their outcomes.* | +  +  +  + | +  +  -  - | +  +  +  + | +  +  +  + | +  +  +  + | +  +  +  + | +  +  +  + |
| 6 | Clinical Findings | *Describe significant physical examination (PE) and important clinical findings.* | + | + | + | + | + | + | + |
| 7 | Timeline | *Historical and current information from this episode of care organized as a timeline (figure or table).* | +/- | - | - | - | + | - | + |
| 8 | Diagnostic Assessment | *Diagnostic methods (PE, laboratory testing, imaging, surveys).*  *Diagnostic challenges.*  *Diagnosis (including other diagnoses considered).*  *Prognostic characteristics when applicable.* | +  +  +  - | +  +  +  - | +  +  +  - | +  +  +  - | +  +  +  - | +  +  +  - | +  +  +  - |
| 9 | Therapeutic Intervention | *Types of therapeutic intervention (pharmacologic, surgical, preventive).*  *Administration of therapeutic intervention (dosage, strength, duration).*  *Changes in therapeutic interventions with explanations.* | +  -  + | +  -  + | +  +  + | +  -  + | +  +/-  + | +  +  + | +  +  + |
| 10 | Follow-up and Outcomes | *Clinician- and patient-assessed outcomes if available.*  *Important follow-up diagnostic and other test results.*  *Intervention adherence and tolerability. (How was this assessed?)*  *Adverse and unanticipated events.* | +  -  -  + | +  +  +  - | +  -  -  - | +  +  -  + | +  +  -  - | +  -  +  + | +  -  -  + |
| 11 | Discussion | *Strengths and limitations in your approach to this case.*  *Discussion of the relevant medical literature.*  *The rationale for your conclusions.*  *The primary “take-away” lessons from this case report (without references) in a one paragraph conclusion.* | -  +  +  + | -  +  +  + | -  +  +  - | +  +  +  + | -  +  +  - | -  +  +  + | -  +  +  + |
| 12 | Patient Perspective | *The patient should share their perspective on the treatment(s) they received.* | - | - | - | - | - | - | - |
| 13 | Informed Consent | *The patient should give informed consent. (Provide if requested.)* | + | - | - | + | + | - | + |
|  |  |  | Letter to the Editor | Letter to the Editor | Case Report | Letter to the Editor | Clinical Letter | Clinical Letter | Case Report |
